# Preparation of a Porous, Sintered and Reaction-Bonded Si_3_N_4_ (SRBSN) Planar Membrane for Filtration of an Oil-in-Water Emulsion with High Flux Performance

**DOI:** 10.3390/ma11060990

**Published:** 2018-06-11

**Authors:** Lin Li, Er-Ze Gao, Hamidreza Abadikhah, Jun-Wei Wang, Lu-Yuan Hao, Xin Xu, Simeon Agathopoulos

**Affiliations:** 1 Chinese Academy of Sciences Key Laboratory of Materials for Energy Conversion, Department of Materials Science and Engineering, University of Science and Technology of China, Hefei 230026, China; lilin123@mail.ustc.edu.cn (L.L.); gez0417@mail.ustc.edu.cn (E.-Z.G.); hamid@mail.ustc.edu.cn (H.A.); wjw@mail.ustc.edu.cn (J.-W.W.); hly@ustc.edu.cn (L.-Y.H.); 2 Materials Science and Engineering Department, University of Ioannina, GR-451 10 Ioannina, Greece; Sagat@cc.uoi.gr

**Keywords:** SRBSN, porous ceramic, membrane filtration, oil–water separation

## Abstract

A porous, sintered, and reaction-bonded Si_3_N_4_ (SRBSN) planar membrane was prepared by phase-inversion tape-casting, nitridation (at 1350 °C), and sintering (at 1650 °C) of silicon slurry. The membrane was comprised of uniform rod-like β-Si_3_N_4_ crystals with a large length/diameter ratio and had high porosity and bending strength. The prepared membrane features a typical asymmetric structure with a skin layer, a sponge layer, and finger-like voids and an average pore size of 0.61 μm. A high permeation flux of 367 L m^−2^ h^−1^ and an oil rejection of 88.6% were recorded in oil-in-water emulsion separation experiments. These results suggest that SRBSN membranes have excellent potential for the treatment of oily wastewater.

## 1. Introduction

The rapid growth in industrial production, such as that in oil and gas petroleum refineries and the pharmaceutical, metallurgical, fertilizer, and petrochemical industries, has inevitably led to an increase in the generation of oily wastewater in the form of an oil-in-water emulsion, which has a concentration that usually ranges between 50 and 1000 mg/L [1,2]. The direct discharge of these emulsions to the environment is obviously not safe for human or aquatic life [3].

Generally, the size of oil droplets in a stable oil-in-water emulsion is smaller than 20 μm. Thus, it is almost impossible to separate the oil phase by methods which merely apply gravity [4,5]. Several technologies, such as coagulation, flocculation, air flotation, chemical de-emulsification, ultrasonic separation, and membrane filtration, are often used for oil removal [1,6]. An emerging technology that has attracted increasing attention in the treatment of oily wastewater is the membrane separation process, as it is environmentally friendly, contributes to energy conservation, and displays high separation efficiency [5,7,8].

Polymer membranes, such as nylon 6, polyvinyl alcohol (PVA), polysulfone (PSf), polyethersulfone (PES), polyvinylidene fluoride (PVDF), polyacrylonitrile (PAN), cellulose acetate (CA), polyphenylenesulfone, and poly(ethylene glycol) diacrylate, have been widely investigated for oil/water separation [2,9,10,11,12,13,14]. On the other hand, ceramic membranes are sufficiently good for industrial applications because they display high selectivity, superior chemical and thermal stability, and, most importantly, a longer lifetime compared to polymeric membranes. Furthermore, ceramic membranes allow for the precise adjustment of the appropriate pore size within a narrow pore size distribution [15], which is an important factor in the separation of oil and water [1,16,17,18,19]. Ceramic membranes, such as alumina, zirconia, titania, silica, silicon carbide, and mullite [3,7,16,17,20,21,22,23], some low-cost ceramic membranes made of kaolin, blast furnace slag, and fly ash [24,25,26], and polymer/ceramic composite membranes [27,28,29,30,31] have already been applied in separation processes for oil and water.

A literature survey shows that β-silicon nitride (β-Si_3_N_4_) porous ceramic membranes, as well as those of its solid solutions (β-SiAlON), have never been investigated in this field. Silicon nitride is an important structural material [32] applied in ball bearings, cutting tools, and hot section components in turbine engines because of its remarkably high strength, fracture toughness, thermal stability, and chemical stability [33]. Interwoven rod-like β-Si_3_N_4_ crystals with a high ratio of length/diameter can ideally form pores with a narrow pore size distribution, resulting in excellent porous ceramic membranes suitable for a separation process. Moreover, Si_3_N_4_ ceramic is nontoxic and helpful in maintaining the natural environment [34,35]. Zhang Jiang-wei et al. produced β-Si_3_N_4_ hollow-fiber ceramic membranes by a combined phase-inversion and sintering method. A good permeate flux and ultrahigh salt rejection were achieved when they were used in a membrane distillation process [36,37]. Wang Jun-wei et al. synthesized β-SiAlON planar ceramic membranes with a similar structure to β-Si_3_N_4_, which also displayed good performance in membrane distillation experiments [38,39].

Nevertheless, in the above studies, the preparation of β-Si_3_N_4_ was quite costly due to the expensive raw material (which is fine α-Si_3_N_4_ powder) and the high sintering temperature (1700 °C). However, sintered and reaction-bonded Si_3_N_4_ (SRBSN) ceramics are popular on account of their lower production cost since cheap raw material (Si) is used and the reaction of Si and N_2_ or NH_3_ takes place at a relatively low temperature (~1300 °C) followed by post sintering at ~1700 °C. Moreover, the final products obtained by the SRBSN method are superior to the Si_3_N_4_ ceramics produced by the direct sintering of Si_3_N_4_ powder (SSN) since they exhibit higher strength and negligible dimensional variations [40,41,42,43].

Phase-inversion tape-casting is a promising molding technology for producing asymmetric membranes with long, straight, finger-like voids across the membrane from the top to the bottom, leaving a skin layer with a thickness of several microns at the top and a spongy layer with a thickness of ~100 μm at the bottom of the membrane [44,45,46,47]. This kind of structure is expected to effectively increase the permeate flux.

This paper reports on the preparation of sintered and reaction-bonded Si_3_N_4_ porous ceramic membranes with a typical asymmetric structure by a combined phase-inversion tape-casting and sintering reaction of Si and NH_3_ method and on the application of the produced membranes in an oil–water separation process. The ceramic membranes are expected to be highly hydrophilic owing to the intrinsic hydrophilicity of Si_3_N_4_ and, therefore, to oppose oil attachment and effectively suppress permeation flux decline, to be highly porous with a narrow pore size distribution, and to exhibit high mechanical strength because of the tightly bonded long rod-like β-Si_3_N_4_ crystals. As far as the cross-flow membrane separation efficiency is concerned, the permeation flux along with the oil rejection at several pressures and feed flow velocities were measured. The ability to recycle the membranes was also evaluated. The results are discussed in order to show that the fabrication of SRBSN porous ceramic planar membranes is an effective way to produce membranes which can provide a highly efficient treatment of oily water.

## 2. Materials and Methods

Commercially available silicon (Si) fine powder (Qinhuangdao Eno High-Tech Development Co., Ltd., Qinhuangdao, China; average particle size 1 μm) was heat treated at 600 °C for 5 h in air. Then, the suspensions needed for applying the SRBSN reaction method were prepared as follows. A well-stirred powder mixture, which contained 46.5 g of the above-mentioned Si powder, 1 g of Al_2_O_3_, and 2.5 g of Y_2_O_3_ (both from Sinopharm Chemical Reagent Co., Ltd., Shanghai, China, >99.99%), with the Al_2_O_3_ and Y_2_O_3_ being used as sintering aids in order to form an intergranular glass phase that can promote the growth of β-Si_3_N_4_ grains, was mixed with 30 mL ethanol and then ball-milled in an alumina jar using high-purity Si_3_N_4_ grinding balls for 12 h. The slurry was dried at 80 °C and sieved through a 200 mesh screen.

The above dried powder was mixed with the dispersant of *O*-(2-aminopropyl)-*O*′-(2-methoxyethyl) -polypropyleneglycol (AMPG) (Mw = 600, Sigma-Aldrich Co., Ltd., St. Louis, MO, USA) and the binder of polyethersulfone (PESf) (Radel A-100, Solvay Advanced Polymers, Brussels, Belgium). The resultant mixture was added to the solvent of *N*-methyl-2-pyrrolidone (NMP) (CP, Sinopharm Chemical Reagent Co., Ltd., Shanghai, China). Slurries with various amounts of dispersant AMPG and solvent NMP were prepared. The detailed compositions of the slurries produced are listed in Table 1. As far as the designation of the samples is concerned, the dispersant was 2, 4, 6, and 8 wt %; hence, these samples are designated as S2A375, S4A375, S6A375, and S8A375, respectively. Moreover, the samples had a solid-loading as 35, 37.5, 40, and 42.5 vol %; thus, the samples are denoted as S4A35, S4A375, S4A40, and S4A425, respectively. For comparison purposes, suspensions with non-heat-treated Si powder were also similarly prepared.

The mixtures were ball-milled in a planetary ball-mill for 24 h to form stable suspensions. Then, the rheological properties of these suspensions were measured at 25 °C with the aid of a rheometer (Model AR2000, TA Corporate, New Castle, DE, USA). According to the results on shear-thinning behavior and viscosity (shown in the Results section), the suspension S4A375 qualified for the next step of tape-casting.

More specifically, the suspension S4A375, after degassing, was cast on a Mylar sheet by the doctor blade method using a blade gap of 1 mm at room temperature and ambient humidity. The obtained tape was soaked in a water bath for 4 h and then dried at room temperature for 12 h. Planar membranes in disk form were cut out of the green body. The disks were placed inside a powder bed, which was composed (in wt %) of a well-mixed powder of 30% boron nitride (BN) and 70% of a well-mingled powder that contained 93% Si_3_N_4_, 5% Y_2_O_3_, and 2% Al_2_O_3_. Nitridation took place at 1350 °C for 4 h, and the sample was named S1350. Sintering was done in a graphite furnace under an atmosphere of flowing N_2_ at several temperatures, i.e., 1600, 1625, 1650, and 1675 °C (the dwelling time at sintering temperature was 4 h and the heating and cooling rates were 5 K/min). The samples were named S1350-1600, S1350-1625, S1350-1650, and S1350-1675, respectively.

The crystalline phases were identified by X-ray diffraction analysis (XRD, Philips PW 1700, Royal Philips, Amsterdam, The Netherlands) using Cu Kα 1 radiation at a scanning rate of 2°/min. The morphology of a membrane’s surface and cross-sections was observed by a scanning electron microscope (SEM, JEOLJSM-6390LA, Tokyo, Japan). The mechanical properties of the membranes were measured with three-point bending strength tests (INSTRON Model 5567, Norwood, MA, USA, with a maximum load cell of 1 kN). The porosity and the pore size distribution of the membranes were measured by the Archimedes and the bubble method, respectively.

Gas and water permeability measurements were conducted in order to qualify the membrane which would be tested in the oil–water separation experiments. The gas (N_2_) permeation flux (Jg) (in L_N2_ m^−2^ h^−1^) was calculated according to Equation (1) by applying several N_2_ pressures on the membrane, and then measurements (by a flow meter) of the volume of the permeated gas at a given time were taken.

**Figure 1:**
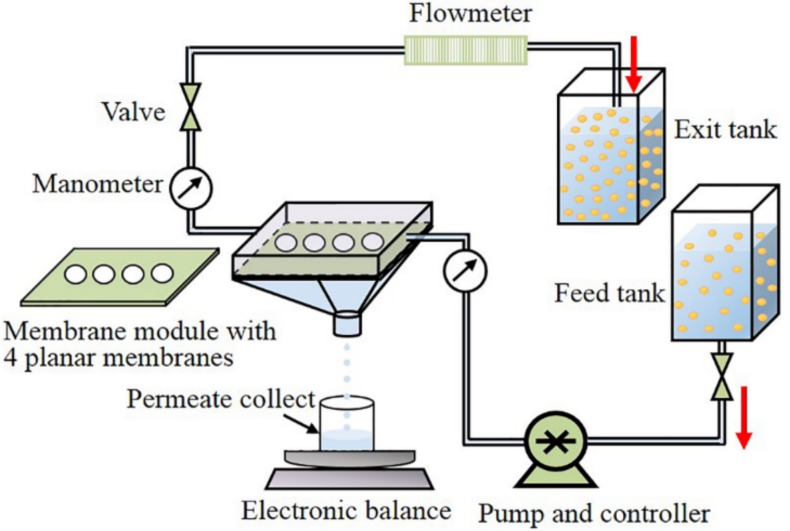
Schematic representation of the cross-flow filtration system used in the microfiltration experiment.

Jo = V/(Ao⋅Δt)(1)

where Jo is the gas permeation flux (Jg), V is the permeated volume during the measured time (Δt), and Ao is the membrane effective area. Water permeability was measured in the apparatus described in the oil–water separation experiments (Figure 1) using pure deionized water instead of the oily emulsion.

The oil–water separation efficiency of the qualified membrane was measured by using an oil-in-water emulsion, which had a concentration of 1000 mg/L and was prepared as follows. Five grams of gasoline and 0.05 g of tween 80 were added to 5 L of deionized water using a mixer homogenizer (Omni International, Kennesaw, GA, USA) at 800 rpm for 15 min at 25 °C. Regarding its coalescence and homogeneity features, the resultant emulsion was stable since there was no evidence of formation of an oil layer at the top of the surface for a period of 1 week. The oil droplet size distribution of the feed emulsion was measured by dynamic light scattering (Zetasizer µV, Malvern Instruments Ltd., Malvern, UK). A BX51 metalloscope (Olympus, Southend, UK) was also employed to observe the oil droplets in the feed emulsion and in the permeated water. The oil concentration was determined by using a UV-vis spectrophotometer (Persee, TU-1810, Beijing, China) at a wavelength of 240 nm where the maximum absorbance was recorded.

The oil–water separation experiments were conducted in a lab-scale cross-flow filtration system (Figure 1). Four membranes, each with an effective diameter of 20 mm, were assembled on the membrane module. The prepared oil-in-water emulsion was flowing over the upper side of the membranes under several pressures (0.05, 0.10, 0.15, and 0.20 bar) and flow velocities (1, 1.5, 2, and 2.5 L/min). The permeate water was collected on the other side of the membrane. The permeate flux (Jp) through the membrane was calculated by the above-mentioned Equation (1), where Jo is the permeate flux (Jp) and V is the permeated water volume. The oil rejection (R%) was calculated by Equation (2):

R% = (Cf − Cp)/Cf(2)

where Cf and Cp are the concentrations of the oil in the feed and the permeated solutions, respectively.

## 3. Results

### 3.1. Rheological Properties of the Suspensions

**Figure 2:**
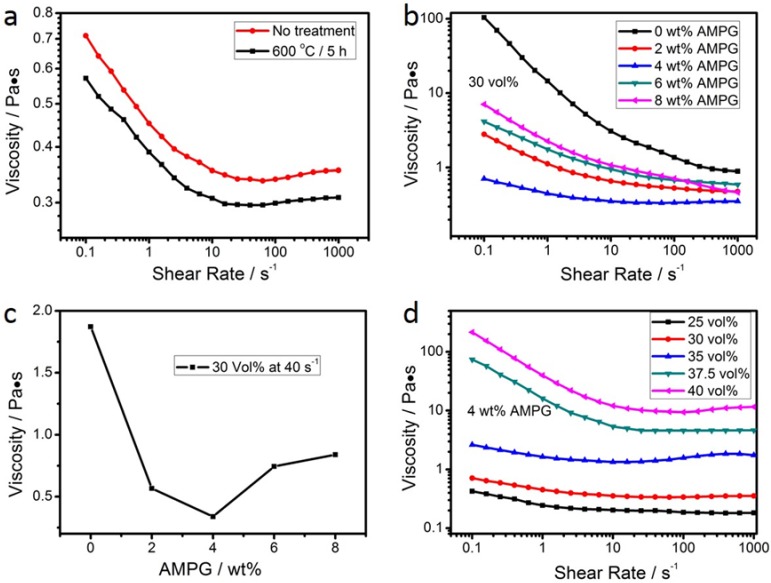
Influence of (**a**) the heat treatment of Si powder, (**b**) the amount of dispersant, and (**d**) the solid-loading on the viscosity measured at a shear rate from 0.1 up to 1000 s^−1^, and (**c**) the influence of the amount of dispersant in the suspensions on the viscosity from (**b**) at a shear rate of 40 s^−1^. (**a**) suspension with different heat treatments of Si powder, original or heat-treated at 600 °C for 5 h, with 30 vol % solid-loading and 4 wt % dispersant. (**b**,**c**): suspensions with heat-treated Si powder with 30 vol % solid-loading. (**d**) suspensions with heat-treated Si powder with 4 wt % dispersant).

The rheological properties of the prepared suspensions are summarized in the plots of viscosity versus shear rate in Figure 2. More specifically, the influence of the heat treatment of Si powder on the viscosity of the produced suspensions with 30 vol % solid-loading and 4 wt % dispersant is shown in Figure 2a. These results suggest that the heat treatment of Si powder at 600 °C for 5 h improves the fluidity of the suspension (i.e., lower viscosity was recorded). This is ascribed to the surface oxidation of Si particles in the powder occurring during the heat treatment. Hence, hereafter, all the experiments refer to suspensions prepared with Si powder and heat-treated at 600 °C for 5 h.

The effect of the amount of dispersant on the rheology properties of the suspensions with 30 vol % solid-loading is presented in the plots of Figure 2b,c, where Figure 2c shows the viscosity measured in different suspensions at a shear rate of 40 s^−1^. All of the suspensions showed a shear thinning behavior (i.e., viscosity decreases when shear rate increases, Figure 2b). The lowest viscosity was recorded in the suspension with 4 wt % AMPG (Figure 2c), which was considered to be the optimal content of AMPG. These results suggest that AMPG is a very effective dispersant for dispersing the starting ceramic powders.

The influence of solid-loading on the viscosity of the suspensions with 4 wt % AMPG is shown in Figure 2d. In the case of a low solid-loading, the suspensions show a near Newtonian behavior with low viscosity. A high solid-loading causes an increase in viscosity, but the effect of shear thinning is more pronounced than in suspensions with a lower solid-loading. Apparently, aggregates were formed in these suspensions, but they collapse when the shear rate increases. Shear thinning and moderate viscosity values for shear rates between 10 and 100 s^−1^ are observed when the solid-loading is 37.5 vol %. These conditions were adopted in the following experiments because both of them are a good match for the conditions in phase-inversion tape-casting.

### 3.2. Characterization of the Membranes

**Figure 3:**
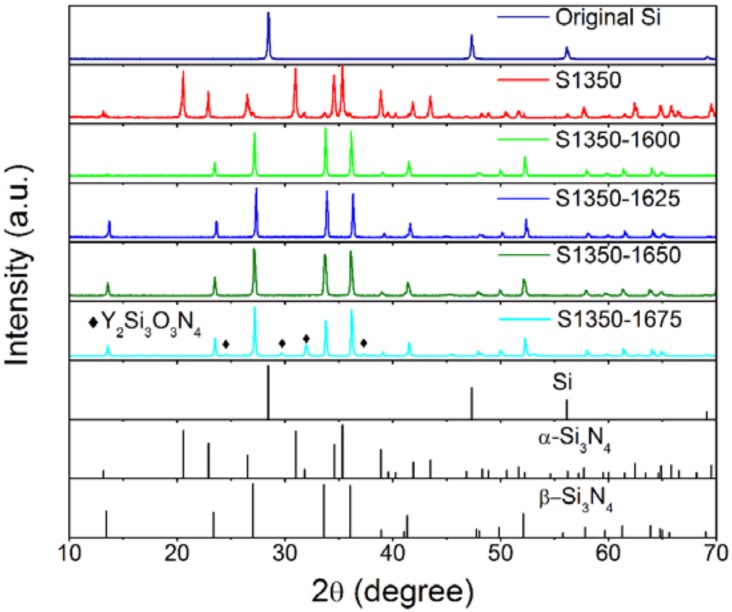
X-ray diffractograms of the produced tapes after sintering at 1600, 1625, 1650, and 1675 °C along with the diffractograms of the original Si powder and the tape that was not subjected to sintering. The standard patterns of Si, α-Si_3_N_4_, and β-Si_3_N_4_ are plotted at the bottom of the diagram.

The above results of the rheology properties qualified the suspension S4A375 for the steps of phase-inversion tape-casting, nitridation at 1350 °C, and sintering. The crystalline phases developed in the produced tapes after sintering at 1600, 1625, 1650, and 1675 °C are shown in the X-ray diffactograms in Figure 3. The diffractograms of the original Si powder and of the tape that was not subjected to sintering are also plotted. It is clearly seen that after nitridation at 1350 °C, α-Si_3_N_4_ was formed, suggesting the effective occurrence of the reaction of the chemical Equation (3):

3Si + 2N_2_ → Si_3_N_4_. (3)

The α-Si_3_N_4_ phase completely transformed into β-Si_3_N_4_ phase after sintering at higher temperatures (≥1600 °C). At the highest sintering temperature tested of 1675 °C, a secondary phase of Y_2_Si_3_O_3_N_4_ was also recorded and attributed to the reaction of silicon nitride with the additive of yttrium oxide [41].

**Figure 4:**
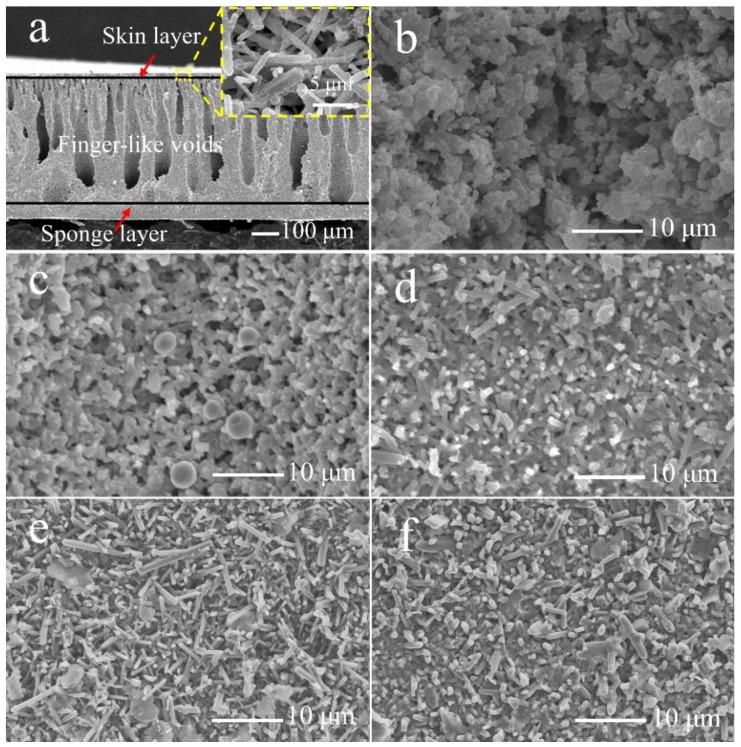
SEM images of the cross-section and surface of the prepared membranes. Typical cross-section of the prepared membranes ((**a**) this image was obtained from the membrane S1350-1650; the inset shows a high magnification of the skin layer) and surface morphology (top-view) of S1350 (**b**); S1350-1600 (**c**); S1350-1625 (**d**); S1350-1650 (**e**); and S1350-1675 (**f**).

The typical asymmetric microstructure was observed in the cross-section of the membranes as shown in Figure 4a (this image was obtained from the membrane S1350-1650, but similar cross-section microstructures were observed in all of the produced membranes). More specifically, a very thin skin layer and a sponge layer (with a thickness of ~100 μm) were formed and the high magnification image of the skin layer is shown in the inset, which shows a porous structure with rod-like crystals intertwined with each other. Between them, the straight finger-like voids zone is approximately 80% of the volume, whereas the sponge-like region occupies approximately the remaining 20% [46,47]. The thickness of the membranes was ~450 μm. It is well-known that this kind of configuration improves the porosity and favors the permeation flux in oil–water separation experiments, where the thin skin layer and the sponge layer function as the separation layers, while the finger-like voids act as mass transfer channels.

The influence of the nitridation and the sintering temperature on the microstructure of the surfaces of the planar membranes is shown in the top-view SEM images in Figure 4b–f, respectively. After nitridation, which completely occurred at 1350 °C (as the XRD analysis confirmed), the angular silicon particles where transformed to sleek α-Si_3_N_4_ grains (Figure 4b). A quintessential rod-like morphology of β-Si_3_N_4_ crystals was observed in the membranes sintered at 1600 °C (Figure 4c). At 1625 °C, the rod-like β-Si_3_N_4_ crystals are interlaced with each other, forming uniform pores (Figure 4d) [42]. At 1650 °C, the elongated rod-like β-Si_3_N_4_ crystals further grew, making the pores more uniform (Figure 4e). Nonetheless, sintering at a higher temperature (1675 °C) resulted in a compact membrane surface, and the porous structure was gradually lost (Figure 4f).

**Figure 5:**
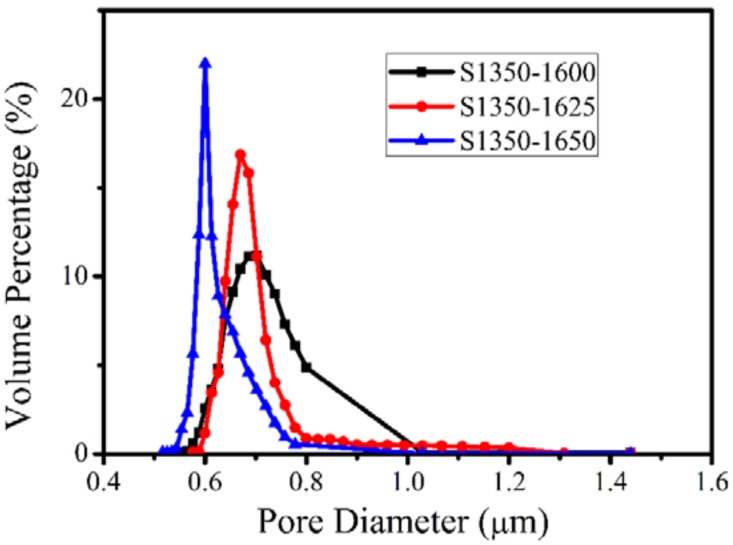
Pore-size distribution of the membranes sintered at various temperatures.

**Figure 6:**
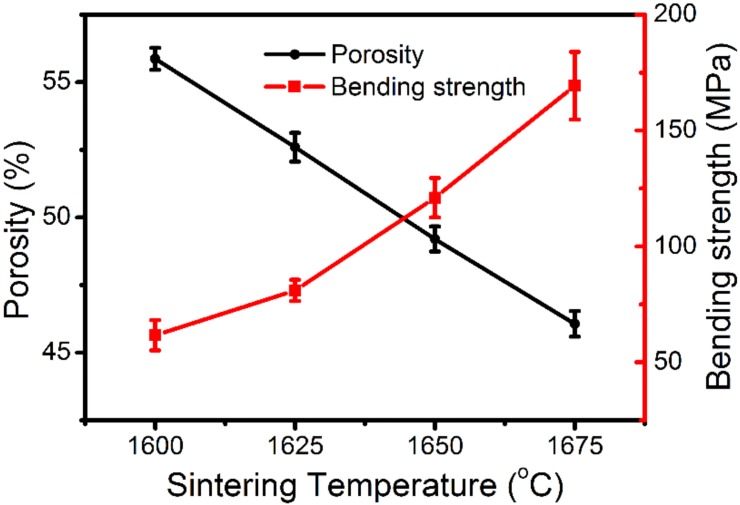
Influence of sintering temperature on porosity and the three-point bending strength of the membranes (the results presented are the average values (and their SD) from five independent measurements).

The effect of the sintering temperature on the pore size is quantitatively represented in the pore-size distribution curves in Figure 5. The average pore size of the membranes S1350-1600, S1350-1625, and S1350-1650 is 0.72, 0.68, and 0.61 μm, respectively; however, the surface of the membrane S1350-1675 is so dense that the pore-size distribution is difficult to measure. The influence of sintering temperature on porosity, as well as on the bending strength of the membranes, which is directly affected also by the porosity (i.e., the higher the porosity, the lower the bending strength), is shown in Figure 6. Porosity ranges between 55.8% and 45.9%. The highest bending strength of 170 MPa, achieved after sintering at 1675 °C, is high enough for a membrane module to scale up practical applications, but it must be disqualified due to very dense microstructure, which is not suitable for filtrating the emulsion. An overall evaluation of all the above properties suggests that the membrane S1350-1650, which has a strength of 130 MPa and a porosity of 48.7%, is the best choice for oil–water separation.

**Figure 7:**
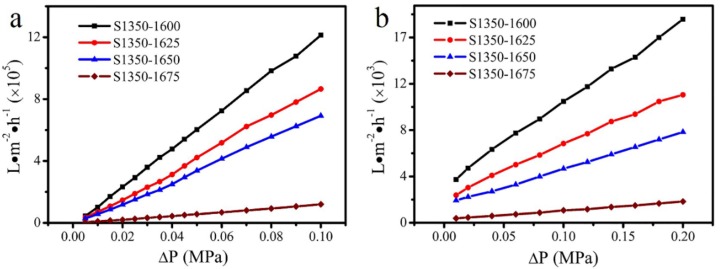
N_2_ (**a**) and water (**b**) permeation properties of the membranes sintered at various temperatures. The *X*-axis is the applied pressure difference between the two sides of a membrane.

Nevertheless, before these experiments, the prepared membranes were also evaluated with respect to their nitrogen and pure water permeation properties and the results are summarized in the plots of Figure 7a,b, respectively. In both cases, the increase of sintering temperatures causes a decrease in gas and water permeation. A very small permeability was recorded in the dense membrane S1350-1675. The highly porous and suitable structure of the membrane S1350-1650 allowed for a huge N_2_ permeation of 6.92 × 10^5^ L m^−2^ h^−1^ and a water permeation of 4.88 × 10^3^ L m^−2^ h^−1^ at a pressure of 0.1 MPa. Accordingly, the membrane S1350-1650, with high porosity, high bending strength, and an appropriate pore-size distribution, meets all the requirements for the oil–water separation tests with the filtration process.

### 3.3. Filtration of Oil-in-Water Emulsion

**Figure 8:**
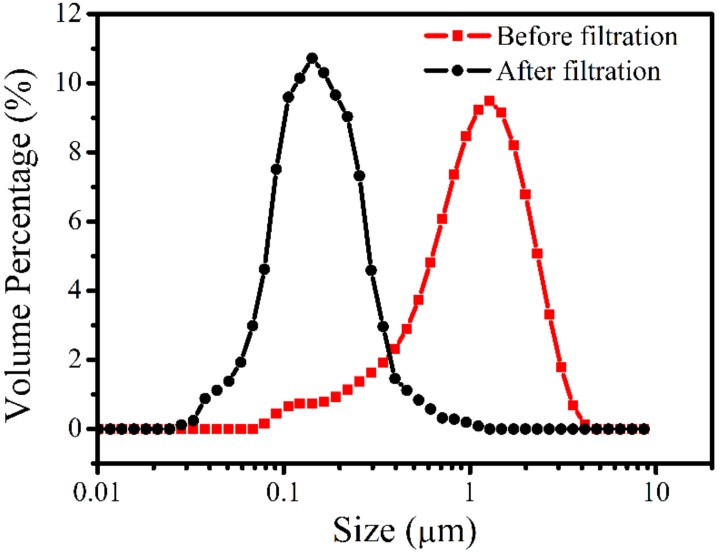
Oil droplets size distributions of the prepared oil-in-water emulsion before and after filtration at 0.1 MPa, 2.5 L/min.

It can be plausibly postulated that in order to purify the oil-in-water emulsion with the intrinsically hydrophilic β-Si_3_N_4_ membrane produced in the present study, the pore diameter of the membrane should be smaller than the size of the oil droplets. The size distribution of the oil droplets in the prepared oil-in-water emulsion, plotted in Figure 8, suggests that their size was in the range 0.07–4 μm, with a peak at ~1.4 μm. In the light of the pore-size distribution curves of Figure 5, this result suggests that the membrane S1350-1650 should efficiently filtrate this oily emulsion. After filtration, the oil droplet size in the permeate water decreased dramatically, confirming the removal of large oil droplets.

**Figure 9:**
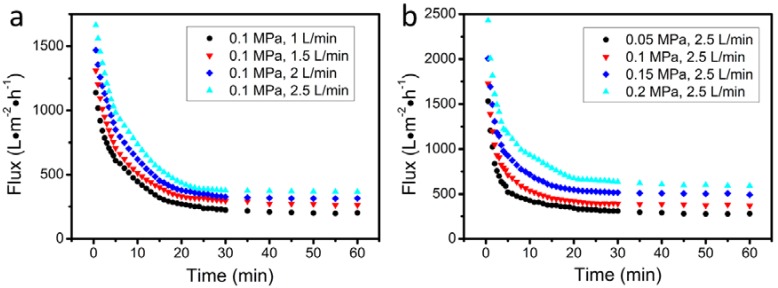
Permeation flux over filtration time for the produced membranes at various (**a**) flow velocities and (**b**) pressing pressures (S1350-1650 membrane, 0.1 MPa, 2.5 L/min).

The effect of flow velocity and applied pressure on the permeation flux was evaluated by using the experimental setup of Figure 1. The results of these experiments are shown in Figure 9a,b, respectively. In all cases, the permeate flux declined rapidly within the first 10 min, attributed to a pore-blocking effect, but then it gradually became steady. The results of these experiments are quantitatively presented in Table 2, where the values of oil rejection are also listed. The results of Table 2 suggest that high pressure and high flow velocity favor the permeation flux. Nonetheless, oil rejection is favored by increasing flow velocity and decreasing pressure.

**Figure 10:**
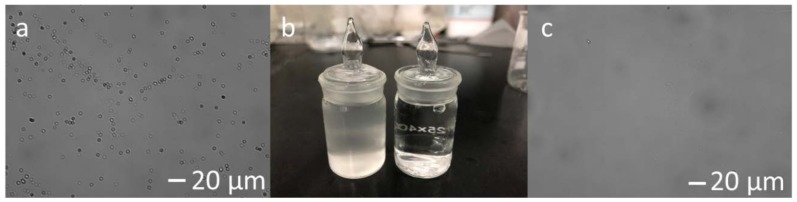
The beaker with oil-in-water emulsion (left-hand side in (**b**)), the beaker with water filtered through the membrane S1350-1650 at 0.1 MPa, 2.5 L/min (right-hand side in (**b**)), and the corresponding images from an optical microscope of the emulsion (**a**) and the filtered water (**c**).

The difference between the emulsion and the water obtained after filtration at 0.1 MPa, 2.5 L/min is shown in the photograph in the middle of Figure 10 (Figure 10b), with the two glass bottles and the corresponding photographs from an optical microscope shown in Figure 10a,c, respectively. It is immediately seen that the feculent emulsion transformed into limpid water after filtration. Oil droplets with a diameter of 1–2 μm were homogeneously dispersed in the emulsion (Figure 10a) and disappeared in the permeated water (Figure 10c).

**Figure 11:**
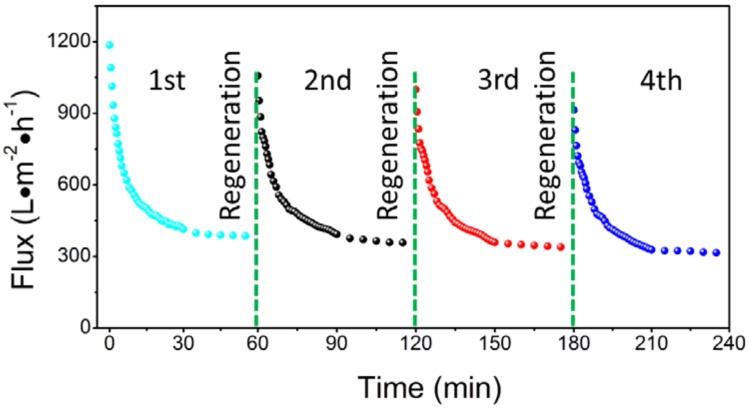
Variation of permeation flux with time during the filtration process (S1350-1650 membrane, 0.1 MPa, 2.5 L/min) of an oil-in-water emulsion when membrane regeneration succeeded via a simple ultrasonic agitation method applied four times.

Membrane fouling, which is a direct result of the interactions between the oil droplets and the membrane surface, causes a decline in flux, which is sometimes irreversible. In the present study, a simple ultrasonic agitation for 5 min was carried out to recover the membrane flux. The results after four runs (each run lasted 1 h and then ultrasonic agitation followed) are shown in Figure 11. After four runs, the permeation flux declined only by 7.5% (from 358 to 331 L m^−2^ h^−1^). This suggests that a simple ultrasonic cleaning is effective to maintain the flux recovery performance of a β-Si_3_N_4_ membrane, which should be attributed to the weak bonding between the hydrophilic Si_3_N_4_ and the hydrophobic oil layer, which can be detached rapidly.

## 4. Discussion

In the process of preparing an appropriate slurry for phase-invasion tape-casting, 4 wt % AMPG showed the best dispersity as shown in Figure 2b,c. Concerning the role of the dispersant, it is suggested that the anchor group of –NH_2_ in the molecule of the AMPG dispersant is adsorbed on the surface of the powder, and accordingly the suspension is stabilized owing to the steric repulsion developed by the polymer chains protruding in the solvent [37]. With low dispersant content, the insufficient coverage of the particle surface with dispersant leads to the formation of agglomerates through the uncovered surface sites due to Brownian motion. When shear stress was applied, the destruction of agglomerates resulted in a decrease of viscosity (i.e., shear thinning). The optimum (plausibly monolayer) dispersant coverage of the particles’ surface was obtained at 4 wt % AMPG, leading to the minimum viscosity of the suspension. In suspensions with a higher content of dispersant, a thicker layer forms on a particle’s surface, and the entanglements among the excess of the dispersant molecules in the solution lead to an increase in viscosity.

In the study, S1350-1650 was chosen to undergo the oil-in-water emulsion filtration because the huge change in pore size between the sintering temperatures of 1650 and 1675 °C made the S1350-1675 surface too dense to be chosen as the separation membrane. Moreover, in comparison with the membranes sintered at lower temperatures, S1350-1650 showed a higher mechanical strength, a smaller pore size, and a narrower pore size distribution. These properties made S1350-1650 the optimum choice for the separation process.

In the oil-in-water emulsion filtration experiments, the effect of pressure and flow velocity on the permeate flux (J_p_) and the oil rejection (R%) should be explained as follows. In the case of a high applied pressure, the oil droplets are settled on the membrane surface and more easily diffused through the pores of the membrane. On the other hand, the increase of flow velocity leads to an increase of the shear stress on membrane surface and, therefore, to a decrease in the height of the sedimentation layer. Finally, the permeation flux increases with the increase of flow velocity. With regard to the increase of oil rejection with the increase of the permeation flux, it might be suggested that the large droplets are more easily removed by the higher shear stress developed at higher flow velocities; thus, fewer oil droplets can be pressed through the membrane and the oil rejection increases [7,48]. The oil droplets that are settled on the membrane surface would block up the pores and finally decrease the permeate flux as shown in Figure 9. Nevertheless, this layer of oil also acts as a dynamic membrane or a barrier against the passage of oil droplets through the membrane to increase the separation efficiency [16]. That is the reason for the high efficiency shown in Table 2, even if the membrane pore size is not much smaller than the oil droplet size.

The results of oil-in-water emulsion filtration experiments in this work are compared with results reported in the literature for other membranes in Table 3. It is immediately concluded that a higher permeation flux was recorded with the membrane prepared in the present study than with most of the other membranes. This is ascribed to the high porosity and the unique membrane structure, which is a result of the phase-inversion tape-casting. The increase in permeation flux is due to the membrane microstructure (Figure 4a), which consists of a finger-like void layer as a supporting layer, a sponge layer, and a skin layer as the functional layer, which makes the separation layer thinner than 100 μm.

## 5. Conclusions

A novel asymmetric, sintered, and reaction-bonded silicon nitride (SRBSN) membrane was fabricated by a phase-inversion tape-casting combined with reaction sintering method. The produced membrane featured high bending strength, high porosity, and an appropriate pore-size distribution ascribed to the growth of fine interlaced β-Si_3_N_4_ crystals. The membrane has a typical asymmetric structure that consists of finger-like voids, which aim to reduce trans-membrane resistance, and sponge and skin layers, which efficiently meet the needs for micro-filtration (MF). Indeed, the produced membrane performed successfully in the filtration of an oil-in-water emulsion, and a higher permeation flux was recorded compared to other membranes reported in the literature. The flux of the membranes is perfectly recovered by a simple ultrasonic agitation method. This good performance is attributed to the hydrophilicity of silicon nitride, which weakens the bonding between the oil droplets and the membrane. Hence, the SRBSN membrane has great prospects for application in the field of oily wastewater treatment.

## Figures and Tables

**Table 1:** Composition of the ceramic slurries (in g).

Sample	Powder Mixture	AMPG	PESf	NMP
S2A375	50	1	7.14	35.61
S4A375	50	2	7.14	35.61
S6A375	50	3	7.14	35.61
S8A375	50	4	7.14	35.61
S4A35	50	2	7.14	39.68
S4A40	50	2	7.14	32.05
S4A425	50	2	7.14	26.12

**Table 2:** Stable permeation flux and rejection (%) of oil from the membrane S1350-1650 at various pressing pressures and flow velocities (it corresponds to Figure 9).

Pressing Pressure (MPa)	Flow Velocity (L min^−1^)	Permeation Flux (L m^−2^ h^−1^)	Rejection (%)
0.1	1	202	81.2
0.1	1.5	262	83.6
0.1	2	316	86.3
0.1	2.5	367	88.6
0.05	2.5	279	91.4
0.1	2.5	367	88.6
0.15	2.5	492	85.8
0.2	2.5	592	83.5

**Table 3:** Comparison of the permeate flux and oil rejection in the present work with other membranes reported in the literature for the separation of an oil-in-water emulsion.

Material	Pore Size (μm)	Porosity (%)	Oil Concentration (mg/L)	Pressure (kPa)	Permeate Flux (L m^−2^ h^−1^)	Oil Rejection (%)	Reference
Si_3_N_4_	0.63	48.7	1000	100	367.0	88.6	This work
M, Q, etc.	1.06	26.0	100	207	199.4	87.0	[6]
M, Q, etc.	2.16	37.4	400	207	79.7	98.5	[49]
M-A	0.29	56.0	1000	300	244.0	81.3	[16]
ZrO_2_/α-Al_2_O_3_	1.00	40.0	5500	100	27.0	94.3	[23]
Fly ash	0.77	-	2000	100	93.0	98.2	[50]
TiO_2_	0.90	43.3	200	207	186.6	99.6	[22]
SiC/Al_2_O_3_	0.07	44.6	600	101	64.1	99.9	[21]

M: mullite; Q: quartz; A: alumina.

## References

[1] Das B., Chakrabarty B., Barkakati P. (2017). Separation of oil from oily wastewater using low cost ceramic membrane. Korean J. Chem. Eng..

[2] Padaki M., Surya Murali R., Abdullah M.S., Misdan N., Moslehyani A., Kassim M.A., Hilal N., Ismail A.F. (2015). Membrane technology enhancement in oil–water separation: A review. Desalination.

[3] Garmsiri E., Rasouli Y., Abbasi M., Izadpanah A.A. (2017). Chemical cleaning of mullite ceramic microfiltration membranes which are fouled during oily wastewater treatment. J. Water Process Eng..

[4] Zhu L., Chen M., Dong Y., Tang C.Y., Huang A., Li L. (2016). A low-cost mullite-titania composite ceramic hollow fiber microfiltration membrane for highly efficient separation of oil-in-water emulsion. Water Res..

[5] Zhou J.-E., Chang Q., Wang Y., Wang J., Meng G. (2010). Separation of stable oil–water emulsion by the hydrophilic nano-sized ZrO_2_ modified Al_2_O_3_ microfiltration membrane. Sep. Purif. Technol..

[6] Vasanth D., Pugazhenthi G., Uppaluri R. (2013). Cross-flow microfiltration of oil-in-water emulsions using low cost ceramic membranes. Desalination.

[7] Zhong J., Sun X., Wang C. (2003). Treatment of oily wastewater produced from refinery processes using flocculation and ceramic membrane filtration. Sep. Purif. Technol..

[8] Nandi B.K., Moparthi A., Uppaluri R., Purkait M.K. (2010). Treatment of oily wastewater using low cost ceramic membrane: Comparative assessment of pore blocking and artificial neural network models. Chem. Eng. Res. Des..

[9] Yi G., Chen S., Quan X., Wei G., Fan X., Yu H. (2018). Enhanced separation performance of carbon nanotube–polyvinyl alcohol composite membranes for emulsified oily wastewater treatment under electrical assistance. Sep. Purif. Technol..

[10] Ge J., Jin Q., Zong D., Yu J., Ding B. (2018). Biomimetic multilayer nanofibrous membranes with elaborated superwettability for effective purification of emulsified oily wastewater. ACS Appl. Mater. Interfaces.

[11] Islam M.S., McCutcheon J.R., Rahaman M.S. (2017). A high flux polyvinyl acetate-coated electrospun nylon 6/SiO_2_ composite microfiltration membrane for the separation of oil-in-water emulsion with improved antifouling performance. J. Membr. Sci..

[12] Liao Y., Tian M., Wang R. (2017). A high-performance and robust membrane with switchable super-wettability for oil/water separation under ultralow pressure. J. Membr. Sci..

[13] Ou R., Wei J., Jiang L., Simon G., Wang H. (2016). Robust thermoresponsive polymer composite membrane with switchable superhydrophilicity and superhydrophobicity for efficient oil-water separation. Environ. Sci. Technol..

[14] Wang X., Xiao C., Liu H., Huang Q., Hao J., Fu H. (2018). Poly(vinylidene fluoride-hexafluoropropylene) porous membrane with controllable structure and applications in efficient oil/water separation. Materials.

[15] Kayvani Fard A., McKay G., Buekenhoudt A., Al Sulaiti H., Motmans F., Khraisheh M., Atieh M. (2018). Inorganic membranes: Preparation and application for water treatment and desalination. Materials.

[16] Abbasi M., Mirfendereski M., Nikbakht M., Golshenas M., Mohammadi T. (2010). Performance study of mullite and mullite–alumina ceramic MF membranes for oily wastewaters treatment. Desalination.

[17] Atallah C., Tremblay A.Y., Mortazavi S. (2017). Silane surface modified ceramic membranes for the treatment and recycling of SAGD produced water. J. Pet. Sci. Eng..

[18] Das B., Chakrabarty B., Barkakati P. (2016). Preparation and characterization of novel ceramic membranes for micro-filtration applications. Ceram. Int..

[19] Masuda T., Asoh H., Haraguchi S., Ono S. (2015). Fabrication and characterization of single phase alpha-alumina membranes with tunable pore diameters. Materials.

[20] Al-Harbi O.A., Mujtaba Khan M., Özgür C. (2017). Improving the performance of silica-based crossflow membranes by surface crystallization for treatment of oily wastewater. J. Aust. Ceram. Soc..

[21] Kim S.C., Yeom H.-J., Kim Y.-W., Song I.-H., Ha J.-H. (2017). Processing of alumina-coated glass-bonded silicon carbide membranes for oily wastewater treatment. Int. J. Appl. Ceram. Technol..

[22] Suresh K., Pugazhenthi G. (2017). Cross flow microfiltration of oil-water emulsions using clay based ceramic membrane support and TiO_2_ composite membrane. Egypt. J. Pet..

[23] Chao Y., Guosheng Z., Nanping X., Jun S. (1998). Preparation and application in oil-water separation of ZrO_2_/α-Al_2_O_3_ MF membrane. J. Membr. Sci..

[24] Hubadillah S.K., Othman M.H.D., Harun Z., Ismail A.F., Rahman M.A., Jaafar J., MuniraJamil S., Mohtor N.H. (2017). Superhydrophilic, low cost kaolin-based hollow fibre membranes for efficient oily-wastewater separation. Mater. Lett..

[25] Mohammadi F., Mohammadi T. (2017). Optimal conditions of porous ceramic membrane synthesis based on alkali activated blast furnace slag using Taguchi method. Ceram. Int..

[26] Rasouli Y., Abbasi M., Hashemifard S.A. (2017). Investigation of in-line coagulation-MF hybrid process for oily wastewater treatment by using novel ceramic membranes. J. Clean. Product..

[27] Da Silva Biron D., Zeni M., Bergmann C.P., dos Santos V. (2017). Analysis of composite membranes in the separation of emulsions sunflower oil/water. Mater. Res..

[28] Cai Y., Chen D., Li N., Xu Q., Li H., He J., Lu J. (2017). Nanofibrous metal–organic framework composite membrane for selective efficient oil/water emulsion separation. J. Membr. Sci..

[29] Wang K., Yiming W., Saththasivam J., Liu Z. (2017). A flexible, robust and antifouling asymmetric membrane based on ultra-long ceramic/polymeric fibers for high-efficiency separation of oil/water emulsions. Nanoscale.

[30] Soares S.F., Rodrigues M.I., Trindade T., Daniel-da-Silva A.L. (2017). Chitosan-silica hybrid nanosorbents for oil removal from water. Colloids Surf. A Physicochem. Eng. Asp..

[31] Liu C.-T., Su P.-K., Hu C.-C., Lai J.-Y., Liu Y.-L. (2018). Surface modification of porous substrates for oil/water separation using crosslinkable polybenzoxazine as an agent. J. Membr. Sci..

[32] Wu J.-M., Zhang X.-Y., Xu J., Gan K., Li J.-L., Li C.-H., Yang J.L., Shi Y.S. (2015). Preparation of porous Si_3_N_4_ ceramics via tailoring solid loading of Si_3_N_4_ slurry and Si_3_N_4_ poly-hollow microsphere content. J. Adv. Ceram..

[33] Riley F.L. (2000). Silicon nitride and related materials. J. Am. Ceram. Soc..

[34] Neumann A., Reske T., Held M., Jahnke K. (2004). Comparative investigation of the biocompatibility of various silicon nitride ceramic qualities in vitro. J. Mater. Sci. Mater. Med..

[35] Dion I., Bordenave L., Lefebvre F., Bareille R., Baquey C. (1994). Physico-chemistry and cytotoxicity of ceramics. J. Mater. Sci. Mater. Med..

[36] Zhang J.-W., Fang H., Hao L.-Y., Xu X., Chen C.-S. (2012). Preparation of silicon nitride hollow fibre membrane for desalination. Mater. Lett..

[37] Zhang J.-W., Fang H., Wang J.-W., Hao L.-Y., Xu X., Chen C.-S. (2014). Preparation and characterization of silicon nitride hollow fiber membranes for seawater desalination. J. Membr. Sci..

[38] Wang J.-W., Li L., Zhang J.-W., Xu X., Chen C.-S. (2016). β-Sialon ceramic hollow fiber membranes with high strength and low thermal conductivity for membrane distillation. J. Eur. Ceram. Soc..

[39] Wang J.-W., Li X.-Z., Fan M., Gu J.-Q., Hao L.-Y., Xu X., Chen C.-S., Wang C.-M., Hao Y.-Z., Agathopoulos S. (2017). Porous β-Sialon planar membrane with a robust polymer-derived hydrophobic ceramic surface. J. Membr. Sci..

[40] Alem A., Drew R.A.L., Pugh M.D. (2014). Sintered reaction-bonded silicon nitride foams with a high level of interconnected porosity. J. Mater. Sci..

[41] Golla B.R., Ko J.W., Kim J.-M., Kim H.-D. (2014). Effect of particle size and oxygen content of Si on processing, microstructure and thermal conductivity of sintered reaction bonded Si_3_N_4_. J. Alloys Compd..

[42] Yao D., Xia Y., Zuo K.-H., Jiang D., Günster J., Zeng Y.-P., Heinrich J.G. (2014). The effect of fabrication parameters on the mechanical properties of sintered reaction bonded porous Si_3_N_4_ ceramics. J. Eur. Ceram. Soc..

[43] Tuyen D.-V., Park Y.-J., Kim H.-D., Lee B.-T. (2009). Formation of rod-like Si_3_N_4_ grains in porous SRBSN bodies using 6Y_2_O_3_–2MgO sintering additives. Ceram. Int..

[44] Ren C., Fang H., Gu J., Winnubst L., Chen C. (2015). Preparation and characterization of hydrophobic alumina planar membranes for water desalination. J. Eur. Ceram. Soc..

[45] Gu J., Ren C., Zong X., Chen C., Winnubst L. (2016). Preparation of alumina membranes comprising a thin separation layer and a support with straight open pores for water desalination. Ceram. Int..

[46] Gu J., Wang J., Li Y., Xu X., Chen C., Winnubst L. (2017). Engineering durable hydrophobic surfaces on porous alumina ceramics using in-situ formed inorganic-organic hybrid nanoparticles. J. Eur. Ceram. Soc..

[47] Lu H., Zhang L., Xing W., Wang H., Xu N. (2005). Preparation of TiO_2_ hollow fibers using poly(vinylidene fluoride) hollow fiber microfiltration membrane as a template. Mater. Chem. Phys..

[48] Vinoth Kumar R., Kumar Ghoshal A., Pugazhenthi G. (2015). Elaboration of novel tubular ceramic membrane from inexpensive raw materials by extrusion method and its performance in microfiltration of synthetic oily wastewater treatment. J. Membr. Sci..

[49] Emani S., Uppaluri R., Purkait M.K. (2014). Cross flow microfiltration of oil–water emulsions using kaolin based low cost ceramic membranes. Desalination.

[50] Fang J., Qin G., Wei W., Zhao X., Jiang L. (2013). Elaboration of new ceramic membrane from spherical fly ash for microfiltration of rigid particle suspension and oil-in-water emulsion. Desalination.

